# Clinical Outcomes and Complication Rates of Crown Restorations with Various Endodontic Posts: A Retrospective Analysis

**DOI:** 10.3390/jfb17020084

**Published:** 2026-02-08

**Authors:** Ali Alenezi, Hanin Alsalhi

**Affiliations:** 1 Department of Prosthetics Dental Sciences, College of Dentistry, Qassim University, Buraidah 51452, Saudi Arabia; 2 Department of Conservative Dental Sciences, College of Dentistry, Qassim University, Buraidah 51452, Saudi Arabia

**Keywords:** retrospective study, crowns, survival rate, nonvital, post-and-core technique

## Abstract

**Objective:** This retrospective study was conducted to evaluate long-term outcomes lcomplication rates of crown restorations supported by different types of endodontic posts and to determine the influence of post material on biological and technical outcomes. **Materials and Methods:** Clinical and radiographic data from 437 crowned teeth retained by fiber, metallic, or custom-made posts were collected at Qassim University Dental Hospital between August and November 2025. Biological (secondary caries, periapical lesions) and technical (debonding, fracture, chipping) complications were recorded. Kaplan–Meier and life-table analyses were used to estimate complication-free survival, and Cox regression was employed to identify significant predictors (α = 0.05). **Results:** The mean observation period was 6.76 ± 4.88 years. The overall complication rate was 56.8%. Crowns restored with fiber posts exhibited the lowest complication rate (40.0%) and the highest 15-year cumulative survival (52%), followed by custom-made (38%) and metallic posts (15%). Fiber posts demonstrated a significantly lower hazard of complications than metal posts (HR = 1.70, *p* = 0.009). Female sex (HR = 1.69, *p* = 0.001) and mandibular location (HR = 1.36, *p* = 0.048) were associated with increased risk. Metal–ceramic crowns showed a protective effect compared to ceramic crowns (HR = 0.56, *p* = 0.001). **Conclusions:** The type of post significantly affected long-term prognosis of crowned endodontically treated teeth. Fiber posts provided the most favorable outcomes by minimizing catastrophic root fractures, while metallic and custom-made posts demonstrated higher complication hazards. Crown material, arch location, and patient factors further influenced survival outcomes.

## 1. Introduction

Restoration of endodontically treated teeth (ETT) with crowns remains a cornerstone of prosthodontic practice, particularly when a substantial coronal tooth structure has been lost due to caries, access preparation, or previous restorations [1]. In such cases, a post-and-core foundation is often required to provide a retention and resistance form for the definitive crown [2]. Moreover, the incorporation of a circumferential ferrule measuring 1.5–2 mm is widely recognized as a critical determinant of the long-term longevity and prognosis of restoration [3].

The most used materials for post fabrication are metals and fiber-reinforced composites [4,5]. Metal posts may either be prefabricated or custom-made, with the cast metal post-and-core regarded as the conventional standard of care [6,7]. Meanwhile, their appears a lack of consensus in the literature regarding standardized treatment protocols, with clinicians selecting various post-and-core materials based on case-related factors [8]. In addition, it has been reported that the use of posts has been reported to effect the biomechanical behavior of the root, leading to complications that may compromise long-term success [9,10].

Biological complications, such as periapical pathology and secondary caries, remain critical concerns. Several cohort studies have reported that recurrent caries is one of the most frequent biological complications following crown placement, especially in situations of poor marginal adaptation or inadequate plaque control [11,12]. Likewise, endodontic failures and periapical lesions may occur if coronal leakage allows bacterial penetration [13]. This underlines the importance of both root canal quality and coronal seals. Gingival inflammation and the loss of periodontal attachment have also been associated with subgingival margins or over-contoured restorations. Moreover, the extensive dentin removal often required for rigid post placement can weaken the root, increasing susceptibility to vertical root fracture (VRF) and non-restorable tooth loss [6]. Post and crown restorations do not directly induce periapical lesions; rather, lesions may develop secondary to coronal leakage, restoration failure, or pre-existing endodontic pathology. Preoperative radiographs should be reviewed when available to exclude teeth with evident periapical pathology at baseline.

Technical complications are equally significant in post-restored teeth. Commonly reported technical failures include post debonding, loss of retention, and post or core fracture [6,14]. Fiber-reinforced composite posts, while biomechanically compatible with dentin, are prone to debonding, particularly when adhesive protocols are compromised [15]. In a systematic review published by Sorrentino et al., most complications observed in ETT supported by fiber posts and single crowns were related to post debonding and loss of retention [16]. Metallic posts, both custom-made and prefabricated, demonstrate reliable retention but can be associated with risks of VRF, which may requires tooth extraction [6,15]. Nevertheless, prefabricated metal posts have been linked to unfavorable outcomes, including non-restorable failures and compromised aesthetics [17]. Zirconia posts offer aesthetic advantages but are associated with unfavorable fracture patterns and are difficult to retrieve in cases of failure [18]. In addition, crown-related technical issues, such as loss of retention or ceramic chipping, are frequently encountered, especially under high occlusal stress [19].

Evidence indicates that the type of post may influence the pattern of failure rather than the absolute incidence of complications [20]. In a systematic review and meta-analysis, Figueiredo et al. reported that fiber posts were associated with fewer catastrophic failures but more debonding than metallic posts [6]. Similarly, Naumann et al. observed that fiber post failures were usually repairable, whereas metallic post failures often resulted in tooth loss [20]. The presence of a ferrule of at least 2 mm has been repeatedly shown to be a decisive factor for prognosis by significantly reducing both biological and technical complications regardless the type of post system [21]. Other factors, such as post length and diameter, residual dentin thickness, cementation technique, occlusal loading, and patient factors, such as parafunctional habits or oral hygiene, also contribute to outcomes [20]. The performance of restorations supported by different types of post materials may vary over time, underlining the multifactorial nature of long-term outcomes [22].

Despite a growing body of evidence, most available studies are prospective trials, in vitro experiments, or meta-analyses [23,24]. Long-term clinical investigations remain scarce, especially studies that compare complication rates across different post-systems in clinical setting. 

Therefore, the aim of this retrospective study was to investigate the complication rate of crown restorations supported by different types of endodontic posts. By assessing biological and technical complications in a large cohort with long-term follow-up, this study seeks to clarify the influence of post type on clinical performance and provide evidence-based guidance for restorative decision-making. We hypothesized that the type of endodontic post is associated with complication-free survival of crowned endodontically treated teeth, with fiber posts demonstrating more favorable long-term outcomes compared with metal-based systems.

## 2. Materials and Methods

The investigation involved the retrospective review of existing clinical and radiographic records of patients who received full-coverage crowns. Furthermore, the investigation received approval from the institutional Research Ethics Committee and was conducted in accordance with the principles outlined in the STROBE (Strengthening the Reporting of Observational Studies in Epidemiology) checklist (Supplementary Table S1) [25]. All procedures involving participants complied with the ethical standards of the institutional and/or national research committee and with the 2024 Declaration of Helsinki. Data were collected from all patients who received post-retained crowns from August 2025 to November 2025 during routine follow-up visits. Because inclusion was limited to patients who attended recall visits during this interval, selection and survivorship biases may be present, and asymptomatic failures or extractions performed elsewhere may be underrepresented.

### 2.1. Eligibility Criteria

Participants were eligible if they were 18 years of age or older and able to provide informed consent. Patients with direct restorations, fixed partial dentures, or removable prostheses were excluded.

### 2.2. Clinical and Radiographic Assessment

Standardized clinical and radiographic evaluations were performed by 2 calibrated clinicians to identify biological and technical complications.

Complications were classified as biological or technical based on predefined diagnostic criteria. Secondary caries was diagnosed when clinical examination and/or bitewing or periapical radiographs demonstrated carious lesions adjacent to crown margins requiring operative intervention. Periapical pathology was assessed radiographically using the standardized long-cone technique. Technical complications included post or crown debonding, material chipping, post fracture, and root fracture. Debonding was defined as partial or complete loss of retention confirmed clinically and documented in patient records, with or without radiographic confirmation. Chipping or fracture of the crown or post was diagnosed based on clinical inspection and radiographic findings, where applicable. Oral hygiene was evaluated with the Simplified Oral Hygiene Index (OHI-S) that categorizes hygiene as good, fair, or poor based on the extent of plaque and calculus [26]. A plaque disclosure solution was used to improve visualization (Garnet 56-00170 Disclosing Solution, ADS, Hialeah, FL, USA). The OHI-S assessments were performed by 2 calibrated clinicians following the criteria of Greene and Vermillion. Periapical radiographs were used because they represent the standard imaging modality available in routine records for retrospective analyses. Caries was defined as radiolucency adjacent to restoration margins with clinical correlation, and periapical pathology as periapical radiolucency consistent with inflammatory lesions. Three-dimensional imaging was not routinely available.

### 2.3. Definitions of Survival and Complications

For survival analyses, the event was defined as the first occurrence of any biological or technical complication. Each tooth was counted only once in the analysis, even if multiple complications developed during follow-up. Teeth without documented complications were censored at the time of the last recorded clinical or radiographic evaluation. Complication-free survival was defined as time to first biological or technical complication; reparable and non-restorable events were analyzed equally, which is acknowledged as a limitation. Only one crown per patient was included in the dataset; therefore, clustering effects were not present. If multiple restorations had been present, this would have required adjusted modeling.

### 2.4. Data Collection Variables

Recorded variables included patient sex, crown location (maxillary or mandibular), restorative material (ceramic, zirconia-based, or metal–ceramic), and type of endodontic post (fiber, metallic, or custom-made). Ceramic crowns were grouped collectively, whereas zirconia-based crowns were analyzed separately.

### 2.5. Statistical Analysis

Analyses were performed using IBM SPSS Statistics software (Version 30; IBM Corp., Armonk, NY, USA). Univariate Cox regression models were applied to evaluate associations between clinical variables and complications, expressed as hazard ratios (HRs) with 95% confidence intervals (CIs). Complication-free survival was estimated with the Kaplan–Meier analysis, defining the event as the first occurrence of any biological or technical complication. Cumulative survival was further assessed using life-table analysis. Intergroup comparisons were conducted with the Mann–Whitney U test, with the level of statistical significance set at α = 0.05. Given the retrospective design, a formal a priori power calculation was not feasible; however, the final sample size was considered adequate for exploratory survival analyses and exceeded those of several comparable retrospective cohorts.

## 3. Results

**Figure 1:**
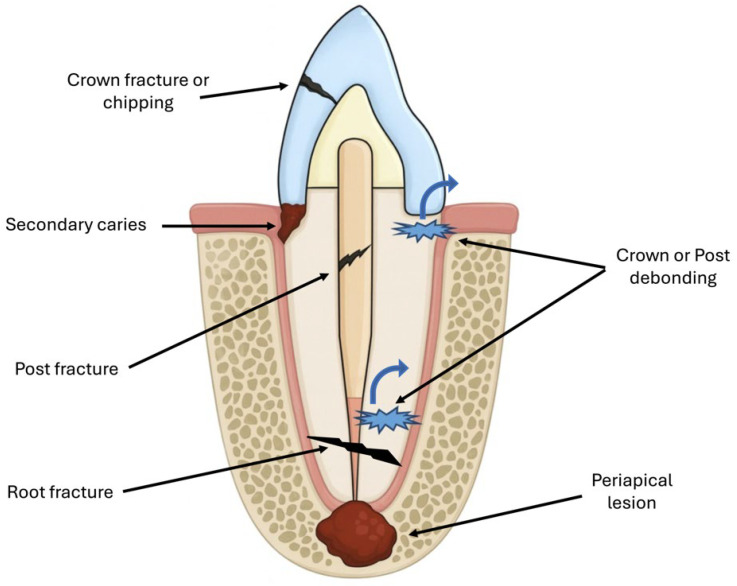
Illustration of the most commonly found complications associated with crown restoration supported by endodontic post. Biological complications included secondary caries and periapical pathology. Technical complications included post or crown debonding, material chipping, post fracture, and root fracture.

**Figure 2:**
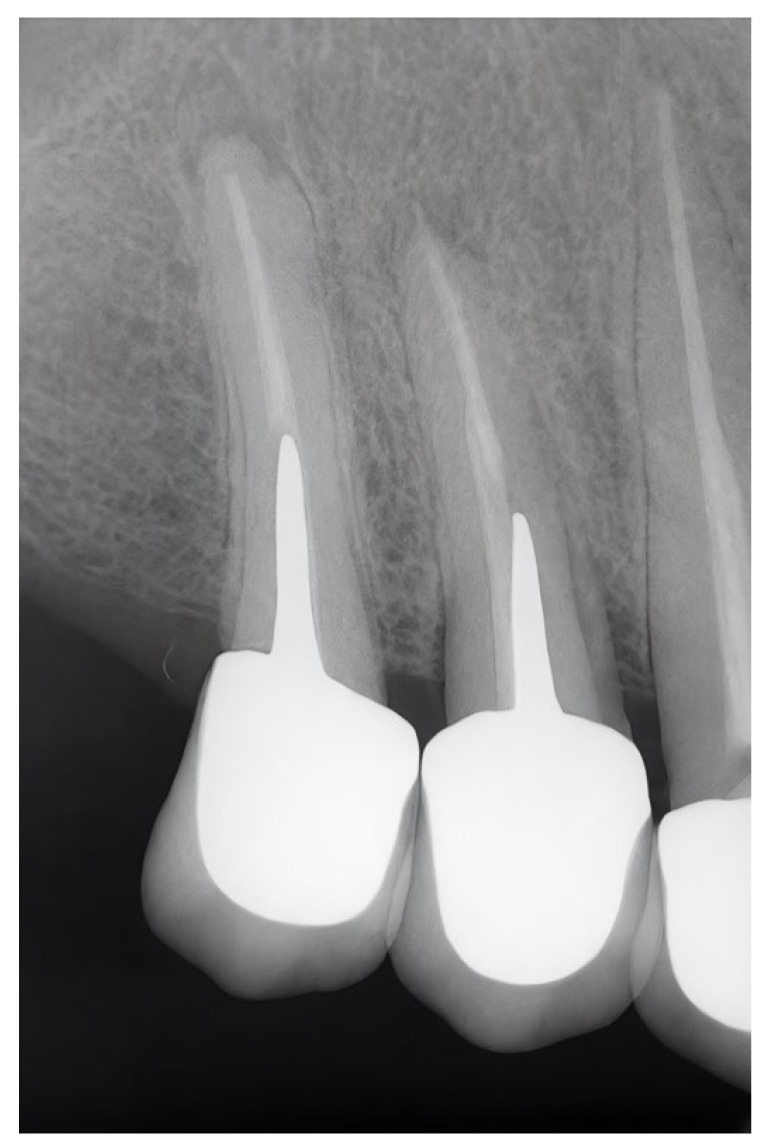
Apical radiograph illustrating ceramic crowns supported by custom-made post-and-core restorations on the upper-right premolars, with evident periapical radiolucency indicative of apical pathology.

A total of 437 crowns were included, with a mean observation period of 6.76 ± 4.88 years (Table 1). Of these, 105 crowns (24.0%) were placed in male patients and 332 crowns (76.0%) in female patients. Complications were observed in 52.4% of the male patients and 58.1% of the female patients. With respect to arch location, maxillary crowns represented 61.3% of the sample and showed complications in 55.2% of cases, whereas mandibular crowns (38.7%) demonstrated a higher complication rate of 59.2%. Anterior crowns presented complications in 61.6% of the cases, compared with 54.3% in posterior crowns. Figure 1 illustrates the complications most commonly found with regard to crown restorations supported by endodontic posts. Figure 2 shows an apical radiograph of ceramic crowns supported by custom-made post-and-core restorations on the upper-right premolars, with evident periapical radiolucency demonstrating signs of apical lesions.

Oral hygiene was strongly associated with outcomes. Patients with poor hygiene (68.6%) exhibited the highest complication rate (62.0%), while those with good hygiene (3.0%) demonstrated significantly fewer complications (15.4%). Meanwhile, cases classified as having good oral hygiene exhibited no biological complications, as shown in Table 1. Regarding post materials, crowns restored with fiber posts showed the lowest overall complication rate (40.0%) compared with metal posts (61.7%) and custom-made posts (50.0%). For crown materials, ceramic crowns demonstrated a complication rate of 45.6%, zirconia crowns 41.5%, and metal–ceramic crowns 62.5%, which was the highest (Table 1).

The life-table survival analysis indicated variable outcomes according to the post material. Crowns restored with fiber posts maintained a cumulative survival rate of 52% after 15 years, while those restored with metal posts decreased to 15% during the same interval. Custom-made posts showed intermediate survival, with a cumulative rate of 38% at 13 years (Table 2, Table 3 and Table 4).

**Figure 3:**
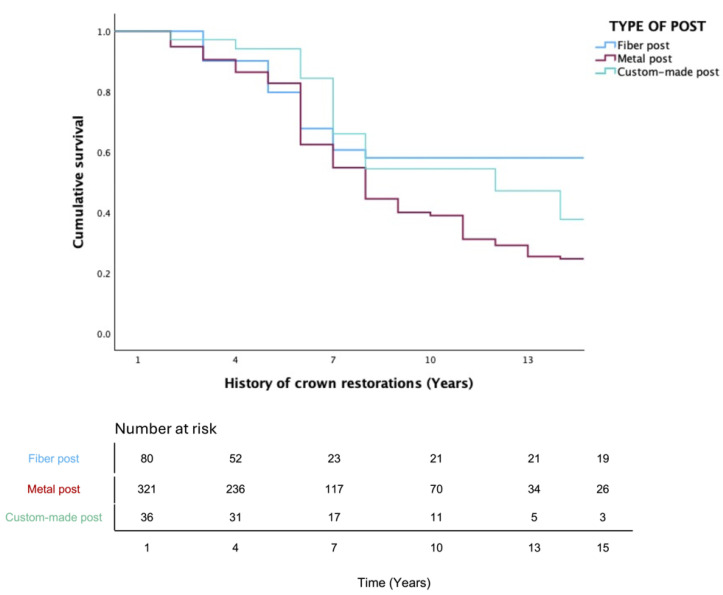
Kaplan–Meier survival function for complication-free survival of crowns based on type of post material (*p* = 0.033). Table at the bottom indicates number at risk.

**Figure 4:**
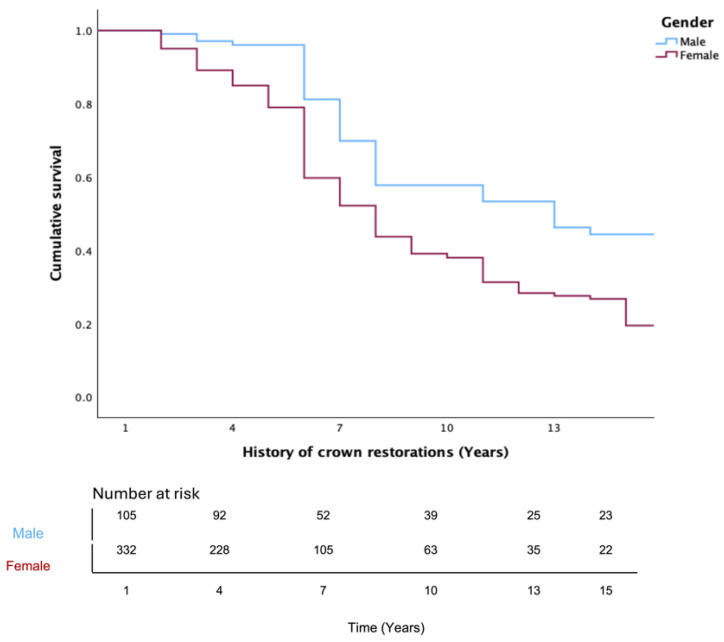
Kaplan–Meier survival function for overall complication-free survival of crowns based on patient’s sex (*p* < 0.001). Table at the bottom indicates number at risk.

**Figure 5:**
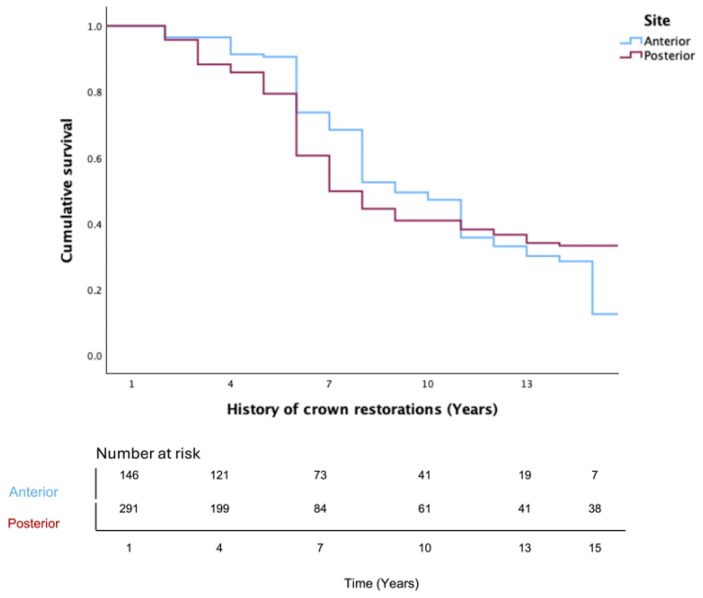
Kaplan–Meier survival function for overall complication-free survival of crowns based on crown location (*p* = 0.033). Table at the bottom indicates number at risk.

These trends were confirmed using the Kaplan–Meier survival functions. Post material had a significant influence on complication-free survival (Figure 3). Fiber posts revealed the most favorable outcomes compared to metal and custom posts (*p* = 0.033). On the other hand, male patients showed significantly higher survival outcomes compared with females (*p* < 0.001; Figure 4). Regarding crown location, maxillary crowns showed significantly higher survival rates than mandibular crowns (*p* = 0.033; Figure 5).

Univariate Cox proportional hazards analysis evaluated several factors (Table 5). Female patients were associated with increased risk (HR = 1.685, 95% CI: 1.225–2.316; *p* = 0.001). Mandibular crowns also carried a higher hazard compared with maxillary crowns (HR = 1.362, 95% CI: 1.003–1.850; *p* = 0.048). Crowns restored with metal posts exhibited a significantly greater risk than fiber posts (HR = 1.700, 95% CI: 1.143–2.529; *p* = 0.009). Meanwhile, metal–ceramic crowns showed a protective effect compared with ceramic crowns (HR = 0.558, 95% CI: 0.400–0.779; *p* = 0.001). Oral hygiene status did not reach statistical significance, although poor hygiene trended toward a higher risk (HR = 1.886; *p* = 0.088). This association should be interpreted as exploratory, as potential confounders such as age, systemic disease, medication use, and caries risk were not available for analysis.

## 4. Discussion

This retrospective investigation sought to clarify the complication rate of crown restorations supported by different endodontic posts over a mean follow-up of nearly 7 years. The results demonstrated clear differences in complication-free survival according to post type, with fiber posts performing most favorably, metal posts showing the highest risk, and custom-made posts presenting intermediate outcomes. Importantly, post material emerged as a statistically significant factor in survival analyses, confirming the hypothesis that the choice of post influences the long-term prognosis of crowned ETT.

The superior clinical outcomes of fiber posts in this study, compared to the other post’s materials, aligns with other clinical and systematic reviews, which have reported that fiber posts are associated with more repairable failures, such as debonding, than root fractures frequently seen with metallic posts. Furthermore, Naumann et al. [20] reported that fiber posts can reduce non-restorable fractures in long-term evaluation, a finding reinforced by Figueiredo et al. [6], who emphasized the shift in failure mode from biological to technical complications. The findings from this study strengthen the existing evidence by confirming, in a large retrospective sample, that the long-term complication risk is significantly higher for metal posts (HR = 1.70; *p* = 0.009), supporting fiber posts as a more biologically conservative choice. This finding corresponds with previous clinical and systematic reviews reporting that metal posts can be associated with a greater risk of root fractures due to their rigidity and stress concentration within the root structure [10,27].

Meanwhile, custom-made posts showed intermediate outcomes. Although they can achieve better adaptation in compromised canals, their rigidity and potential to concentrate stresses may explain the moderate complication rates observed. Furthermore, Naumann et al. and Santos-Filho et al., reported that custom-made posts achieved acceptable survival rate but at the cost of irreparable failure risk [28,29]. On the other hand, fiber posts tend to fail through debonding, which is more biologically favorable and allows for retreatment [15]. For that, while custom-made posts remain a practicable treatment choice in anatomically challenging cases, their long-term prognoses may be compromised by the risk of severe technical and biological complications.

The findings demonstrated that the mode of failure is as important as survival itself. Since their modulus of elasticity is closer to that of dentin, fiber posts dissipate stress more evenly and preserve root integrity, while metal posts may be predisposed to vertical fractures [18]. However, the debonding of crown restorations and fiber posts have been commonly reported as complications. Despite the reportedly inferior bond strength of fiber post surfaces compared to metal posts, fiber posts continue to be selected frequently in clinical practice due to their favorable handling and restorative characteristics [2]. This underlines the clinical decision-making standard that the preservation of tooth structure and ferrule design remains more important than absolute post-survival rates.

The retrospective analysis also revealed significant associations with sex and arch location. Female patients presented a higher hazard of complications (HR = 1.69; *p* = 0.001), although this may reflect systemic or behavioral confounders, such as medication-induced xerostomia, caries risk, or recall compliance, rather than sex itself. Mandibular crowns carried a greater risk than maxillary crowns (HR = 1.36; *p* = 0.048), which is consistent with prior evidence suggesting that increased functional loading in the mandible predisposes restorations to technical complications, such as crown fracture and loss of retention.

While basic complication rates were highest in metal–ceramic crowns, the adjusted analysis revealed a protective effect compared with ceramic crowns (HR = 0.56; *p* = 0.001). This aligns with established evidence that metal–ceramic crowns, particularly in high-load posterior sites, continue to exhibit superior long-term stability compared with some ceramic systems [30]. Zirconia and lithium disilicate systems have demonstrated promising performance in recent years, but their outcomes remain dependent on material and design [31]. Moreover, they may not fully replicate the strength of metal–ceramic restorations under heavy occlusion.

Overall, these findings strengthen the fact that the choice of post material is critical in the long-term success of crowned ETT. Fiber posts appear advantageous by reducing catastrophic failures, particularly when an adequate ferrule is present and adhesive protocols are followed. Meanwhile, metal posts, while still in use for complex cases requiring custom cores, should be used with caution, given their higher complication risks. With respect to material selection, metal–ceramic restorations remain a reliable option in high-stress areas, while ceramic systems can be considered when aesthetics is prioritized and case selection is favorable. Finally, patient-related factors, including oral hygiene and recall compliance, continue to play an important role in reducing biological complications.

This study supports the emerging paradigm that post selection should prioritize failure mode over longevity, consistent with evidence from contemporary reviews and long-term clinical studies [32,33]. The relatively high complication rate (56.8%) may be attributed to the high prevalence of poor oral hygiene and the older age distribution of many cases. Furthermore, the study demonstrates that retrospective data can explain practical survival patterns but must be interpreted cautiously, given the influence of unmeasured confounders, such as parafunctional habits, ferrule height, and adhesive protocols. The ferrule effect is recognized as a critical determinant of the biomechanical behavior and prognosis of ETT [3]. However, in the present retrospective study, ferrule presence and height were not consistently documented and therefore could not be analyzed. In addition, the information regarding crown fabrication process, luting cement types was not consistently documented in the clinical records and therefore could not be analyzed. Furthermore, restorations were performed by several operators within a university-based clinical environment. Variability in these procedural factors may have contributed to the observed differences in complication rates and represents an inherent limitation of this retrospective study. For statistical analysis, Multivariable Cox modeling was carefully considered but it was not applied due to substantial missing data for several clinically relevant covariates, which could have introduced bias and reduced model stability. Given the retrospective nature of this study, the detected differences in survival among post systems should be interpreted as associations rather than causal relationships. Unmeasured clinical variables, including biomechanical and patient-related factors, may have influenced outcomes. Prospective clinical studies with standardized outcome definitions will further strengthen evidence on how the type of endodontic post interacts with the crown material and patient risk factors. Future investigations including finite element analysis could provide valuable biomechanical insights into stress distribution associated with different post systems and help contextualize clinical findings.

## 5. Conclusions

The type of endodontic post can significantly influence the complication rate of crown restorations. Fiber posts demonstrated better long-term outcomes compared to metal posts. Meanwhile, metal–ceramic crowns remained a durable restorative option, particularly under high occlusal loads, although patient-related factors, such as oral hygiene also contributed to complication risk. These findings support the careful selection of post and crown materials to improve the longevity of crown restorations.

## Figures and Tables

**Table 1:** Descriptive data of the crowns included in this study, with follow-up duration between the different factors.

Group		Number of Crowns (%)	Number of Crowns (%) with Signs of Biological Complications	Number of Crowns (%) with Signs of Technical Complications	Total Number of Crowns (%) with Complication	Mean Observation Period (Years) ± SD
Sex	Male	105 (24.0%)	47 (44.8%)	8 (7.6%)	55 (52.4%)	6.76 ± 5.02
	Female	332 (76.0%%)	181 (54.5%)	24 (7.2%)	193 (58.1%)	6.76 ± 4.89
Location	Maxillary	268 (61.3%)	134 (50.0%)	19 (7.1%)	148 (55.2%)	6.76 ± 5.83
	Mandibular	169 (38.7%)	94 (55.6%)	13 (7.7%)	100 (59.2%)	6.79 ± 4.88
Site	Anterior	146 (33.4%)	79 (54.1%)	13 (8.9%)	90 (61.6%)	6.74 ± 4.89
	Posterior	291 (66.6%)	149 (51.2%)	19 (6.5%)	158 (54.3%)	6.76 ± 4.89
Oral hygiene	Good	13 (3.0%)	0 (0.0%)	2 (15.4%)	2 (15.4%)	6.79 ± 4.91
	Fair	124 (28.4%)	50 (40.3%)	18 (14.5%)	60 (48.4%)	6.75 ± 4.88
	Poor	300 (68.6%)	178 (59.3%)	12 (4.0%)	186 (62.0%)	6.77 ± 4.92
Post material	Fiber post	80 (18.3%)	30 (37.5%)	5 (6.3%)	32 (40.0%)	6.81 ± 4.93
	Metal post	321 (73.5%)	181 (56.4%)	25 (7.8%)	198 (61.7%)	6.73 ± 4.89
	Custom-made post	36 (8.2%)	17 (47.2%)	2 (5.6%)	18 (50.0%)	6.77 ± 4.95
Type of material	Ceramic	68 (15.6%)	27 (39.7%)	5 (7.4%)	31 (45.6%)	6.82 ± 4.94
	Zirconia	65 (14.9%)	23 (35.4%)	7 (10.8%)	27 (41.5%)	6.81 ± 4.88
	Metal-ceramic	304 (69.6%)	178 (58.6%)	20 (6.6%)	190 (62.5%)	6.74 ± 4.92
Total		437 (100%)	228 (52.2%)	32 (7.3%)	248 (56.8%)	6.76 ± 4.88

**Table 2:** Life-table survival analysis exhibiting the cumulative survival rate of the complications associated with crowns restored with fiber posts.

Interval Start Time	Number Entering Interval	Number Withdrawing During Interval	Number Exposed to Risk	Number of Terminal Events	Proportion Surviving	Cumulative Proportion Surviving at End of Interval	Std. Error of Cumulative Proportion Surviving at End of Interval
0	80	0	80	0	1.00	1.00	0.00
1	80	3	79	0	1.00	1.00	0.00
2	77	11	72	7	0.90	0.90	0.04
3	59	5	57	0	1.00	0.90	0.04
4	54	4	52	6	0.88	0.80	0.05
5	44	8	40	6	0.85	0.68	0.06
6	30	3	29	3	0.89	0.61	0.07
7	24	2	23	1	0.96	0.58	0.07
8	21	0	21	0	1.00	0.58	0.07
9	21	0	21	0	1.00	0.58	0.07
10	21	0	21	0	1.00	0.58	0.07
11	21	0	21	0	1.00	0.58	0.07
12	21	0	21	0	1.00	0.58	0.07
13	21	0	21	0	1.00	0.58	0.07
14	21	0	21	0	1.00	0.58	0.07
15	21	5	19	2	0.89	0.52	0.08

**Table 3:** Life-table survival analysis exhibiting the cumulative survival rate for the complications associated with crowns restored with metal posts.

Interval Start Time	Number Entering Interval	Number Withdrawing During Interval	Number Exposed to Risk	Number of Terminal Events	Proportion Surviving	Cumulative Proportion Surviving at End of Interval	Std. Error of Cumulative Proportion Surviving at End of Interval
0	321	0	321	0	1.00	1.00	0.00
1	321	15	314	16	0.95	0.95	0.01
2	290	8	286	13	0.95	0.91	0.02
3	269	12	263	12	0.95	0.86	0.02
4	245	18	236	10	0.96	0.83	0.02
5	217	26	204	50	0.75	0.62	0.03
6	141	4	139	17	0.88	0.55	0.03
7	120	6	117	22	0.81	0.45	0.03
8	92	5	90	9	0.90	0.40	0.03
9	78	0	78	2	0.97	0.39	0.03
10	76	13	70	14	0.80	0.31	0.03
11	49	6	46	3	0.93	0.29	0.03
12	40	1	40	5	0.87	0.25	0.03
13	34	1	34	1	0.97	0.25	0.03
14	32	0	32	6	0.81	0.20	0.03
15	26	0	26	7	0.73	0.15	0.03

**Table 4:** Life-table survival analysis displaying the cumulative survival rate for the complications associated with crowns restored with custom-made posts.

Interval Start Time	Number Entering Interval	Number Withdrawing During Interval	Number Exposed to Risk	Number of Terminal Events	Proportion Surviving	Cumulative Proportion Surviving at End of Interval	Std. Error of Cumulative Proportion Surviving at End of Interval
0	36	0	36	0	1.00	1.00	0.00
1	36	1	36	1	0.97	0.97	0.03
2	34	1	34	0	1.00	0.97	0.03
3	33	1	33	1	0.97	0.94	0.04
4	31	0	31	0	1.00	0.94	0.04
5	31	4	29	3	0.90	0.84	0.06
6	24	2	23	5	0.78	0.66	0.09
7	17	0	17	3	0.82	0.54	0.09
8	14	0	14	0	1.00	0.54	0.09
9	14	0	14	0	1.00	0.54	0.09
10	14	6	11	0	1.00	0.54	0.09
11	8	1	8	1	0.87	0.47	0.11
12	6	0	6	0	1.00	0.47	0.11
13	6	2	5	1	0.80	0.38	0.12

**Table 5:** Univariate Cox proportional hazard analyses for complication rate of crown restorations supported by different types of endodontic posts. Reference categories (HR = 1) were male sex, maxillary location, anterior site, good oral hygiene, fiber post, and ceramic crown.

Group		Hazard Ratio (95% CI)	*p*-Value
Sex	Male	1	0.001
	Female	1.685 (1.225–2.316)	
Location	Maxillary	1	0.048
	Mandibular	1.362 (1.003–1.850)	
Site	Anterior	1	0.521
	Posterior	0.905 (0.668–1.226)	
Oral hygiene	Good	1	
	Fair	1.873 (0.450–7.802)	0.388
	Poor	1.886 (0.909–3.911)	0.088
Post material	Fiber post	1	
	Metal post	1.700 (1.143–2.529)	0.009
	Custom-made post	0.838 (0.495–1.416)	0.508
Crown material	Ceramic	1	
	Zirconia	1.224 (0.717–2.090)	0.46
	Metal-ceramic	0.558 (0.400–0.779)	0.001

## Data Availability

The raw data supporting the conclusions of this article will be made available by the corresponding author upon request.

## Supplementary Materials

The following supporting information can be downloaded at https://www.mdpi.com/article/10.3390/jfb17020084/s1: Table S1: STROBE Statement—Checklist of items that should be included in reports of observational studies.

## Abbreviations

The following abbreviations are used in this manuscript:

ETT	Endodontically treated teeth
